# Detection of antibiotic-resistant canine origin *Escherichia coli* and the synergistic effect of magnolol in reducing the resistance of multidrug-resistant *Escherichia coli*

**DOI:** 10.3389/fvets.2023.1104812

**Published:** 2023-03-15

**Authors:** Yin-Chao Tong, Yi-Ning Zhang, Peng-Cheng Li, Ya-Li Cao, Dong-Zhao Ding, Yang Yang, Qing-Yi Lin, Yi-Nuo Gao, Shao-Qiang Sun, Yun-Peng Fan, Ying-Qiu Liu, Su-Zhu Qing, Wu-Ren Ma, Wei-Min Zhang

**Affiliations:** ^1^College of Veterinary Medicine, Northwest A&F University, Yangling, China; ^2^College of Animal Science and Technology, Northwest A&F University, Yangling, China; ^3^Xi'an Veterinary Teaching Hospital, Northwest A&F University, Xi'an, China

**Keywords:** canine, MDR *E. coli*, drug-resistance detection, magnolol, antibiotics adjuvant

## Abstract

**Background:**

The development of antimicrobial resistance in the opportunistic pathogen *Escherichia coli* has become a global public health concern. Due to daily close contact, dogs kept as pets share the same *E. coli* with their owners. Therefore, the detection of antimicrobial resistance in canine *E. coli* is important, as the results could provide guidance for the future use of antibiotics. This study aimed to detect the prevalence of antibiotic-resistance of canine origin *E. coli* in Shaanxi province and to explore the inhibition effect of magnolol combined with cefquinome on MDR E. coli, so as to provide evidence for the use of antibiotics.

**Methods:**

Canine fecal samples were collected from animal hospitals. The *E. coli* isolates were separated and purified using various indicator media and polymerase chain reaction (PCR). Drug-resistance genes [*aacC2, ant(3')-I, aph(3')-II, aac(6')-Ib-cr, aac(3')-IIe, bla*_*KPC*_*, bla*_*IMP*−4_*, bla*_*OXA*_*, bla*_*CMY*_*, bla*_*TEM*−1_*, bla*_*SHV*_*, bla*_*CTX*−*M*−1_*, bla*_*CTX*−*M*−9_*, Qnra, Qnrb, Qnrs, TetA, TetB, TetM, Ermb*] were also detected by PCR. The minimum inhibitory concentration (MIC) was determined for 10 antibiotics using the broth-microdilution method. Synergistic activity of magnolol and cefquinome against multidrug-resistant (MDR) *E. coli* strains was investigated using checkerboard assays, time-kill curves, and drug-resistance curves.

**Results:**

A total of 101 *E. coli* strains were isolated from 158 fecal samples collected from animal hospitals. MIC determinations showed that 75.25% (76/101) of the *E. coli* strains were MDR. A total of 22 drug-resistance genes were detected among the 101 strains. The *bla*_*TEM*−1_gene exhibited the highest detection rate (89.77%). The TetA and Sul gene also exhibited high detection rate (66.34 and 53.47%, respectively). Carbapenem-resistant *E. coli* strains were found in Shangluo and Yan'an. Additionally, in MDR *E. coli* initially resistant to cefquinome, magnolol increased the susceptibility to cefquinome, with an FICI (Fractional Inhibitory Concentration Index) between 0.125 and 0.5, indicating stable synergy. Furthermore, magnolol enhanced the killing effect of cefquinome against MDR *E. coli*. Resistance of MDR *E. coli* to cefquinome decreased markedly after treatment with magnolol for 15 generations.

**Conclusion:**

Our study indicates that antibiotic-resistance *E. coli* has been found in domestic dogs. After treatment with magnolol extracted from the Chinese herb Houpo (*Magnolia officinalis*), the sensitivity of MDR *E. coli* to cefquinome was enhanced, indicating that magnolol reverses the resistance of MDR *E. coli*. The results of this study thus provide reference for the control of *E. coli* resistance.

## 1. Introduction

*Escherichia coli* is one of the most important and common Gram-negative bacteria (GNB) living in the gut of humans and animals which can lead to severe diarrhea. As an opportunistic pathogen, *E. coli* can be transmitted between humans and animals, especially between pets and their owners (1–4). It is therefore important to monitor antimicrobial resistance in *E. coli* derived from pet animals in order to prevent the further development of resistance. Research to identify ways to eliminate bacterial antimicrobial resistance has thus become high priority worldwide (5).

Several systematic reviews have described the complex mechanisms leading to antibiotic resistance, which can be mediated by plasmids, changes in target sites, modifications of antibiotic-degrading enzymes, cell adaptation, and efflux pumps, all of which have been linked to the inappropriate use of antibiotics (6, 7). Thus, the identification of antibiotic alternatives or synergistic approaches to reduce resistance is of clinical importance. Some studies have indicated that combinations of Chinese herb extracts and antibiotics show synergistic effects against *E. coli via* different mechanisms. A range of volatile oils from Cukangchai [*Mallotus philippensis* (*Lam*.) *Muell*. *Arg*.] inhibit conjugal transfer of drug-resistance plasmids, which reduces the lateral transmission of drug-resistance (8). Quercetin has the ability to cause MDR *E. coli* to regain susceptibility to tetracycline by increasing cell permeability and the intracellular drug concentration (9). Two studies found that baicalin from Huangqin (*Scutellaria baicalensis Georgi*) inhibits the activity of NDM-1 and decreases the expression of *fimB*, which is a major bacterial adhesion factor (10, 11). Resveratrol from Lilu (*Veratrum nigrum L*.) reduces the expression of the efflux pump protein AcrAB-TolC in *E. coli* to inhibit drug-resistance (12).

Magnolol is an extractive from Houpo (*Magnolia officinalis*), which was first recorded in the *Shennong Herbal Classic*. As shown in previous studies, magnolol has multiple biological activities, including the prevention and amelioration of diseases such as cancer (13), anti-depressant (14) and anti-diabetes (15) effects, and improvement of growth performance (16). However, the *Ben Cao Gang Mu (Compendium of Materia medica)* indicates gastrointestinal tract diseases such as dysentery and cholera as indications for Houpo, which suggests that Houpo interacts with the intestinal flora. Another study suggested that magnolol affects *E. coli* (17). However, the potential effectiveness of magnolol in the treatment of bacterial infection–related diseases has not been fully explored. In this study, based on a previous synergistic study of magnolol and meropenem (18), magnolol and cefquinome were hypothesized to inhibit *E. coli* synergistically at a safe dose (19). Canine fecal samples were collected from veterinary hospitals to test the antimicrobial resistance of *E. coli* isolates and the potential for magnolol to reverse drug resistance.

This study aimed to detect the prevalence of antibiotic-resistance of canine origin *E. coli* in Shaanxi province and to explore the inhibition effect of magnolol combined with cefquinome on MDR *E. coli*, so as to provide evidence for the use of antibiotics.

## 2. Materials and methods

### 2.1. Sample collection

In this cross-sectional study, 158 canine fecal samples were collected (Supplementary Table 1) from 12 animal hospitals in eight prefecture-level cities (Yulin, Yan'an, Xi'an, Xianyang, Baoji, Ankang, Shangluo, Hanzhong and Weinan) in Shaanxi Province during November 2021 to August 2022, which were numbered by the first letter of the sampling city with patient number.

### 2.2. Bacterial isolation and identification

All collected samples were enriched in trypticase soy broth for 10~12 h at 37°C until reaching logarithmic phase and then transferred onto MacConkey agar and then eosin-methylene blue agar and incubated aerobically for 16~18 h at 37°C. The red isolates on MacConkey agar an the black with metallic luster isolates were chosen and saved from next steps. The *E. coli* isolates were subjected to Gram stain followed by primary identification. All media were purchased from Qingdao Hope Biotechnology Co., Qingdao, China. The *E. coli* strain ATCC^®^ 25922™ preserved in our laboratory was used as a control.

### 2.3. Molecular confirmation of *E*. coli isolates

Single, pure isolates were enriched for a second time in Mueller-Hinton broth (MHB) for 24 h at 37°C. Thereafter, 1 mL of bacterial culture was centrifuged at 14,000 rpm for 15 min. After decanting the supernatant, the pelleted cells were washed with sterile ultrapure water, and the centrifugation and wash steps were repeated twice. To extract genomic DNA, washed bacteria were boiled in sterile ultrapure water for 10 min. After centrifugation at 14,000 rpm for 15 min, the resulting supernatant was used as the DNA template for polymerase chain reaction (PCR) assays (4, 20) using primers specific for 16S rDNA to identify the isolates, as described previously (21, 22). PCR assays were performed in a final volume of 20 μL, consisting of 10 μL of master mix (Dining, China), 1 μL of each forward and reverse primer, 1 μL of DNA template, and 7 μL of nuclease-free water. PCR assays were performed in a thermocycler (Bioer TC-XP-G, China) using the following proGram: initial denaturation at 94°C for 5 min, followed by 30 cycles of denaturation at 94°C for 30 s, annealing (Supplementary Table 2) for 30 s, and extension at 72°C for 30 s, followed by a final extension at 72°C for 7 min. The positive and negative controls were *E. coli* ATCC^®^ 25922™ and nuclease-free water, respectively. Electrophoresis was performed on a 1.5% agarose gel stained with DiRed Safe DNA DYE (Dining, China) to determine the size of PCR products compared to a 2,000-bp DNA ladder. The gel was scanned using a UV-light transilluminator (72/BR04467, Bio-Rad, USA). Confirmed isolates were stored at −80°C in MHB containing 35% glycerol until further analysis.

### 2.4. Antibiotic susceptibility testing

Broth-microdilution assays were performed to determine the antibiotic susceptibility and minimum inhibitory concentrations (MICs) for 10 antibiotics, including ceftiofur, cefquinome, ceftazidime, ceftriaxone, meropenem, norfloxacin, ciprofloxacin, gentamycin, kanamycin and amikacin (Shanghai Macklin Biochemical Co., Ltd, China) as recommended by the Clinical and Laboratory Standards Institute and the European Committee on Antimicrobial Susceptibility Testing. The above antibiotics are commonly used in veterinary clinical treatment of *E. coli* infection. Susceptibility to ceftiofur and cefquinome was determined in reference to previous research (23). All drugs were diluted 2-fold in MHB and mixed with an equal volume of bacterial suspension in a 96-well microtiter plate. Each test was repeated three times. *Escherichia coli* ATCC^®^ 25922™ was used as the quality-control strain (without magnolol). According to a previous report, bacteria resistant to ≥3 different classes of antibiotics were considered MDR (24).

### 2.5. Molecular detection of antibiotic resistance genes

All primers were reflected in Table 1. PCR assays were performed in a final volume of 20 μL, consisting of 10 μL of master mix (Dining, China), 1 μL of each forward and reverse primer, 1 μL of DNA template, and 7 μL of nuclease-free water. PCR assays were performed in a thermocycler (Bioer TC-XP-G, China) using the following proGram: initial denaturation at 94°C for 5 min, followed by 30 cycles of denaturation at 94°C for 30 s, annealing (Supplementary Table 2) for 30 s, and extension at 72°C for 30 s, followed by a final extension at 72°C for 7 min. The negative control was nuclease-free water. Electrophoresis was performed on a 1.5% agarose gel stained with DiRed Safe DNA DYE (Dining, China) to determine the size of PCR products compared to a 2,000-bp DNA ladder. The gel was scanned using a UV-light transilluminator (72/BR04467, Bio-Rad, USA). Confirmed isolates were stored at −80°C in MHB containing 35% glycerol until further analysis.

**Table 1:** Detection rate of drug-resistance genes (*n* = 101).

**Gene**	**Detection rate (%)**	**Gene**	**Detection rate (%)**
*bla_*TEM*−1_*	89.77 ± 0.22	*aac(6')-Ib-cr*	22.77 ± 0.00
*TetA*	66.34 ± 0.00	*TetB*	17.82 ± 0.00
*Sul*	53.47 ± 0.00	*aph(3')-II*	15.84 ± 0.00
*aac(3)-IIe*	41.91 ± 0.22	*QnrB*	8.91 ± 0.00
*bla_*CTX*−*M*−1_*	40.59 ± 0.00	*ErmB*	6.93 ± 0.00
*TetM*	30.69 ± 0.00	*QnrA*	6.27 ± 0.22
*bla_*CTX*−*M*−9_*	30.03 ± 0.22	*bla_*SHV*_*	4.95 ± 0.00
*QnrS*	27.72 ± 0.00	*CMY*	4.95 ± 0.00
*aph(6)-Id*	25.74 ± 0.00	*OXA*	3.96 ± 0.00
*ant(3”)-I*	25.74 ± 0.00	*IMP-4*	3.96 ± 0.00
*aacC2*	23.76 ± 0.00	*bla_*KPC*_*	0.99 ± 0.00

### 2.6. Checkerboard assay

The combined antibacterial effect of magnolol and cefquinome was assessed using a checkerboard assay, as previously described (25). Briefly, both magnolol (≥98% HPLC, Shanghai Aladdin Bio-Chem Technology Co., China) and cefquinome were diluted to prepare seven gradient concentrations ranging from 1/16 MIC to 2 MIC. Each longitudinal column of tubes contained the same concentration of drug A, and each horizontal row of tubes contained the same concentration of drug B. Each tube was inoculated with bacterial suspension to a final density of approximately 1 × 10^6^ CFU/mL. Single-drug control tubes and blank control tubes were also prepared, and *E. coli* ATCC^®^ 25922™ was used as a sensitivity control strain. Six isolates with the highest number of antibiotic-resistance genes were used as experimental bacteria. All tubes were incubated at 37°C for 16 h under aerobic conditions. The experiment was repeated in triplicate. Fractional inhibitory concentration index (FICI) was calculated according to the following formula: FICI = MIC of magnolol in combination/MIC of magnolol alone + MIC of cefquinome in combination/MIC of cefquinome alone. An FICI value ≤ 0.5 indicated synergy; 0.5 < FICI ≤ 0.75 indicated partial synergy; 0.76 < FICI ≤ 1 indicated additive effect; 1 < FICI ≤ 4 indicated indifferent effect; and FICI > 4 indicated antagonism. In this study, synergy and partial synergy were defined as a synergistic relationship, whereas additive, indifferent, and antagonistic results were regarded as a non-synergistic relationship (26).

### 2.7. Time-kill curves

Time-kill assays were used to evaluate the antibacterial effects of the combination of magnolol and cefquinome against MDR *E. coli* by measuring the reduction in the calculated population in CFU/mL within 24 h. Magnolol and cefquinome were incubated with an equal volume of *E. coli* culture at different levels of magnolol and cefquinome (27). As a control, MHB was added instead of magnolol or cefquinome. All samples were cultivated at 37°C. After 0, 2, 4, 6, 8, and 24 h of incubation, 100-μL samples were removed. After dilution to proper levels, 100 μL of each sample was spread onto Mueller-Hinton agar for colony counting. Each assay was repeated in triplicate.

### 2.8. Drug-resistance curves

Drug-resistance curves were used to evaluate the effects of magnolol in reducing the resistance of MDR *E. coli* to cefquinome by determining the MIC after magnolol treatment within 15 generations. Magnolol (0.25 MIC) was incubated with an equal volume of each *E. coli* culture in MHB at 37°C for 16 h. An inoculating loop of each MHB culture was then streaked onto Mueller-Hinton agar and incubated at 37°C for 16 h. After 0, 1, 2, 3, 6, 9, and 15 generations, a single, pure-colony of each isolate was removed and placed in MHB and incubated at 37°C for 16 h, after which the MIC was determined. Each assay was repeated in triplicate.

### 2.9. Statistical Analysis

Data are expressed as the mean ± standard deviation. The statistical significance of differences was determined using *t*-tests with SPSS 27.0 software. For all comparisons, *P* < 0.01 and *P* < 0.05 were considered indicative of statistical significance. All figures were made by GraphPad Prism 8.0.1. The maps were downloaded from Standard Map Service (http://bzdt.ch.mnr.gov.cn/) and edited by Photoshop 2021.

## 3. Results

### 3.1. Samples and *E. coli* isolates

**Figure 1:**
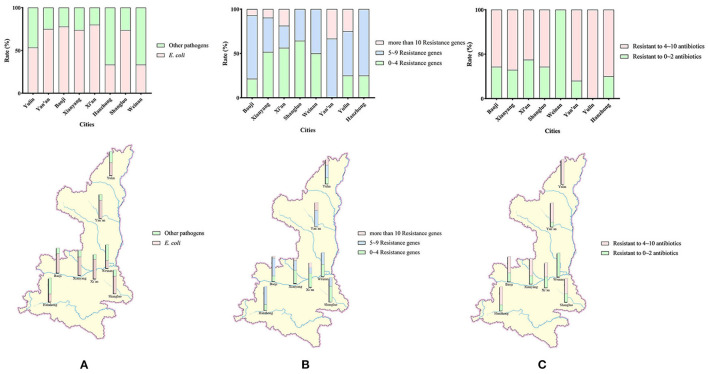
**(A)** Geographical distribution of the sampling and detection rates of *E. coli* isolates. Sources of 158 samples and rates of *E. coli* detected in 8 cities in Shaanxi Province. **(B)** Detection rates and regional distribution of *E. coli* isolates carrying different numbers of antibiotical genes. **(C)** Detection rates and regional distribution of MDR *E. coli* isolates.

A total of 101 *E. coli* strains were isolated from 158 canine fecal samples (Supplementary Table 1) obtained using anal swabs, for a rate of 63.92% (Figure 1A). As shown in Supplementary Figure 1A, 22 drug-resistance genes, including 6 aminoglycoside-resistance genes, 8 β-lactam–resistance genes, 3 quinolone-resistance genes, 3 tetracycline-resistance genes, 1 sulfa drug-resistance gene and 1 macrolide-resistance gene were detected.

**Figure 2:**
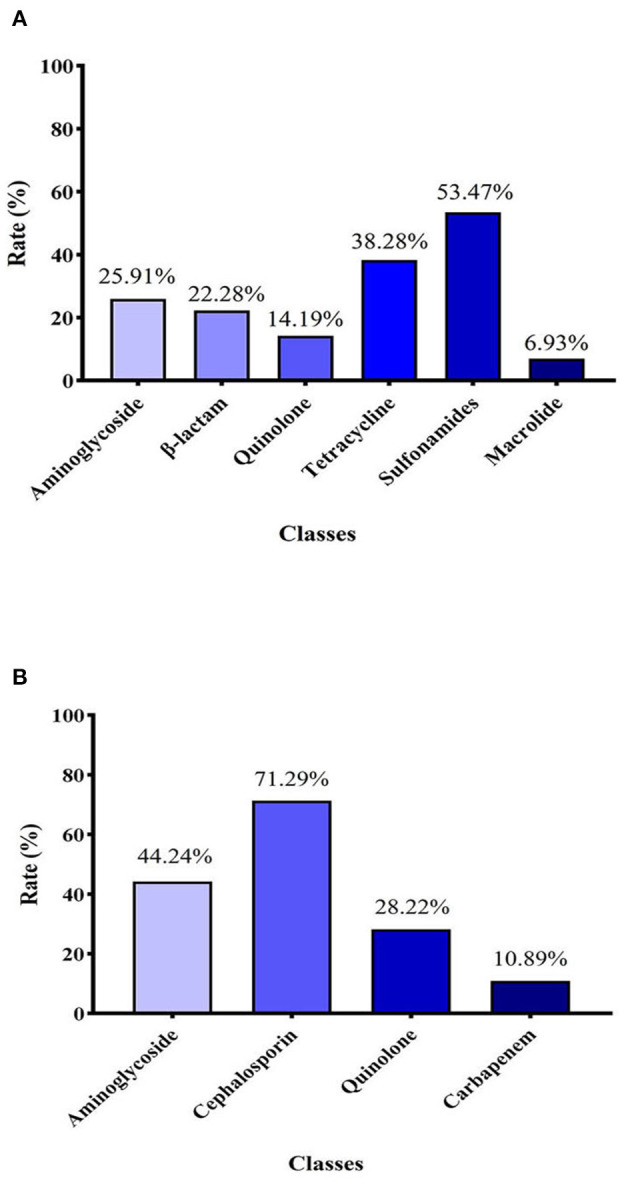
**(A)** Detection rate of 6 classes of antibiotical genes among 101 *E. coli* strains identified. **(B)** Resistance rates of *E. coli* strains to four different classes of antibiotics.

In general, as shown in Figure 2A, the detection rate of sulfa drug–resistance genes was the highest of six types of tested genes, at 53.47%. The detection rate of tetracycline-resistance genes was 38.28%, which was the second highest. The detection rates of aminoglycoside-resistance genes and β-lactam–resistance genes were similar, at 25.91% and 22.28, respectively. The detection rates of quinolone-resistance and macrolide-resistance genes were 14.19% and 6.93, respectively.

In terms of single genes, as shown in Table 1, all tested genes were detected, with *bla*_*TEM*−1_ exhibiting the highest detection rate, at 89.77 ± 0.22%. The detection rates of *TetA* and *sul* were both >50.00%, at 66.34 and 53.47%, respectively. The detection rates of *aac(**3**)-Iie* and *bla*_*CTX*−*M*−1_were similar, at 41.91 ± 0.22% and 40.59%, respectively. The detection rates of *aacC2, ant(3')-I, aph(3')-II, aac(6')-Ib-cr, aph(**6**)-Id, bla*_*CTX*−*M*−9_, *QnrS*, and *TetM* were similar (22.77%~30.69%), followed by *TetB* and *aph(3')-II* (17.82 and 15.84%, respectively). The detection rates of *KPC, IMP-4, OXA, CMY, bla*_*SHV*_*, QnrA, QnrB* and *ErmB* were all < 10% (0.99~8.91%), and the detection rate of *bla*_*KPC*_was the lowest, at 0.99%.

As the result of the high detection rate of *bla*_*TEM*−1_, the resistance rates for extended-spectrum β-lactamases were high. *E. coli* strains with more than 10 resistance genes accounted for 13.86% (14/101) of isolates in this study (Shaanxi Province). As shown in Figure 1B, the geographical rate was 25.00% in Yulin, 18.75% in Xi'an, 9.682% in Xianyang, and 7.14% in Baoji. No isolates with more than 10 resistance genes were detected in Shangluo, Weinan, and Hanzhong. The rates of *E. coli* strains with 5~9 resistance genes were 46.53% in Shaanxi Province, and geographically, 75.00% in Hanzhong, 73.33% in Yan'an, 71.43% in Baoji, 50% in Yulin, 50.00% in Weinan, 38.71% in Xianyang, 35.71% in Shangluo, and 25% in Xi'an.

### 3.2. Antibiotic susceptibility testing

The 10 tested antibiotics were grouped into 4 classes (Supplementary Figure 1B), including cephalosporins (ceftiofur, cefquinome, ceftazidime, ceftriaxone), carbapenems (meropenem), quinolones (norfloxacin, ciprofloxacin), and aminoglycosides (gentamycin, kanamycin, and amikacin). As shown in Figure 2B, the carbapenem class of antibiotics had the lowest resistance rate (10.89%). In contrast, the resistance rate of the cephalosporin class of antibiotics (71.29%) was highest. The resistance rates of aminoglycosides and quinolones were 42.24 and 28.22%, respectively.

In general, the rate of MDR *E. coli* was 75.25% (76/101). The highest rate of MDR *E. coli* occurred in Yulin, at 100%. However, no MDR *E. coli* were detected in Weinan. The detection rate of MDR *E. coli* in other cities was between 56.25 and 80.00%, as shown in Figure 1C.

### 3.3. Synergistic effect of magnolol in combination with cefquinome

**Figure 3:**
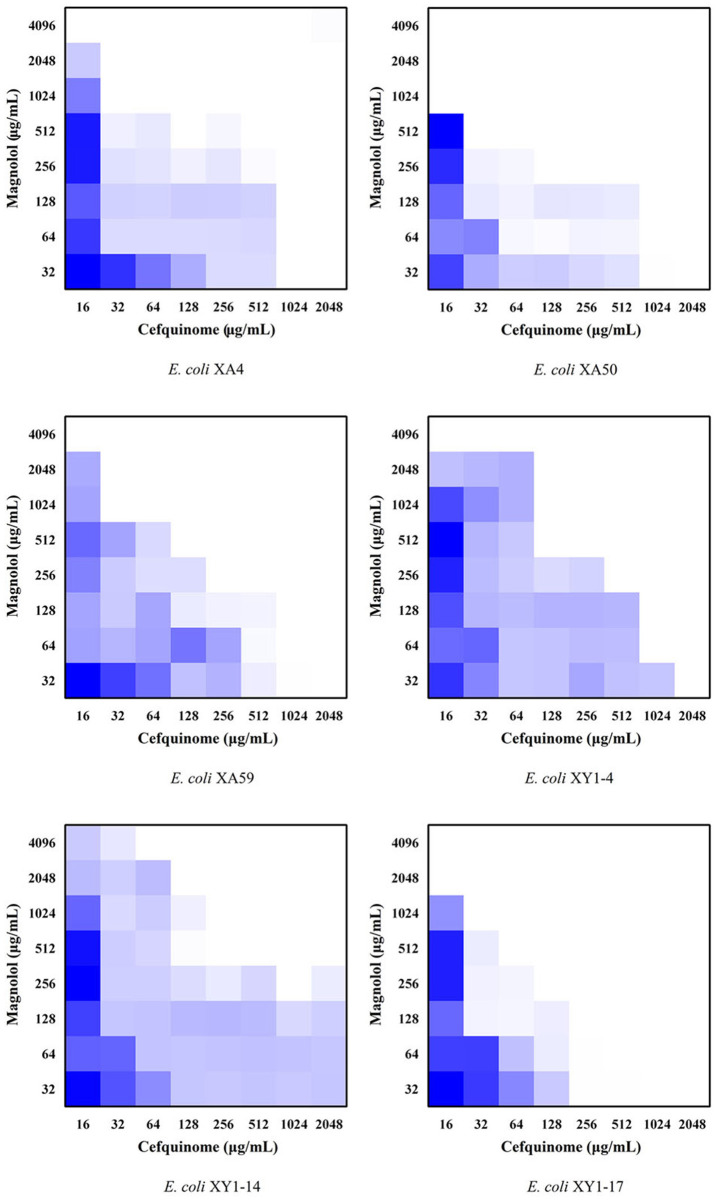
Checkerboard broth assays for magnolol and cefquinome against 6 MDR *E. coli* strains.

To evaluate the potential synergistic effect of magnolol combined with cefquinome, checkerboard dilution assays were performed against 6 MDR *E. coli* strains with the highest number of antibiotic-resistance genes in this study (XA4, XA50, XA 59, XY1-4, XY1-14, and XY1-17). The MIC values of magnolol monotherapy against isolates was shown in Table 2. Notably, as shown in Figure 3 and Table 2, the FICI of ATCC^®^ 25922^TM^ was <0.75, indicating the partial synergistic effect. The FICI of XA4, XA 59, XY1-4 and XY1-14 was <0.5, indicating the synergistic effect. The FICI of XA50 and XY1-17 was <0.75, indicating the partial synergistic effect. What's more, as shown in Figure 3, the use of cefquinome in combined treatment decreased 8- to 32-fold compared to monotherapy, which suggesting that magnolol decreases the resistance of MDR *E. coli* to cefquinome.

**Table 2:** Results of MIC (magnolol) and FICI (magnolol × cefquinome) for seven *E. coli* isolates.

**Strain**	**MIC (μg/mL)**	**FICI**	**Outcome**
ATCC^®^ 25922^TM^	426.67 ± 147.80	0.54 ± 0.19	Partial Synergy
XA4	853.33 ± 295.60	0.28 ± 0.024	Synergy
XA50	1365.33 ± 591.21	0.5 ± 0.13	Partial Synergy
XA59	3413.33 ± 1182.41	0.38 ± 0.12	Synergy
XY1-4	3413.33 ± 1182.41	0.22 ± 0.054	Synergy
XY1-14	6826.67 ± 2364.83	0.18 ± 0.065	Synergy
XY1-17	2730.67 ± 1182.41	0.58 ± 0.14	Partial Synergy

### 3.4. Time-kill analyses

**Figure 4:**
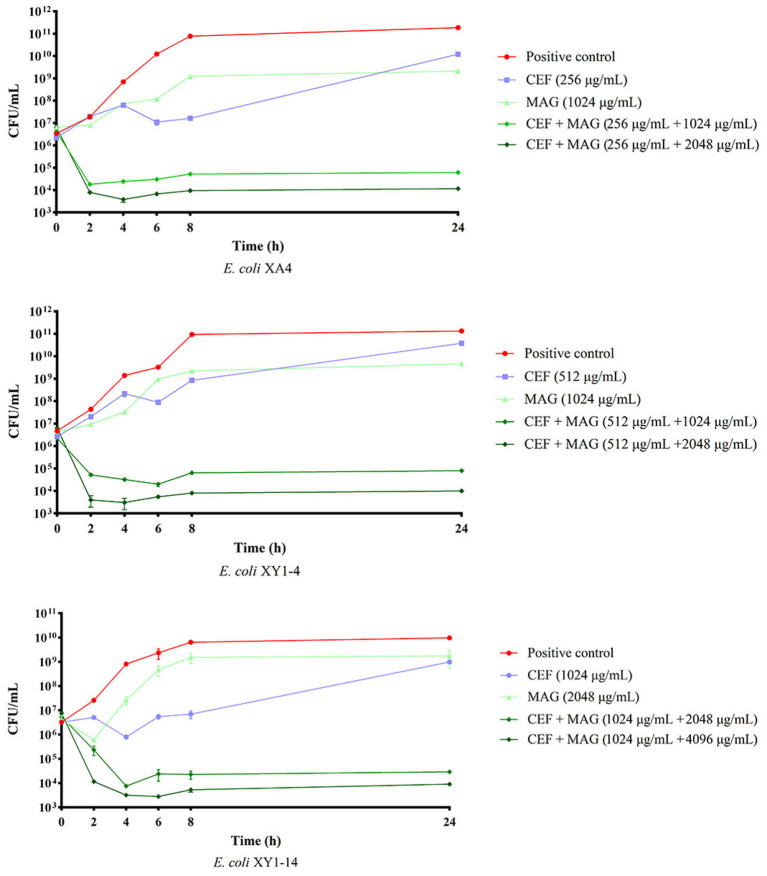
Bactericidal effect of magnolol (MAG) and cefquinome (CEF) in different combinations or levels against MDR *E. coli*. All data are expressed as mean ± SD determined from three independent experiments performed in triplicate.

Based on the results of MIC assays, time-kill curves were prepared to evaluate the bactericidal effect of cefquinome against MDR *E. coli* treated with magnolol. As shown in Figure 4, compared with either the single cefquinome group or the single magnolol group, at all concentrations tested, the combination of magnolol and cefquinome exhibited an enhanced bactericidal effect against the three tested MDR *E. coli* strains within 24 h. During 0–8 h, the population of *E. coli* strains in MAG + CEF group decreased 10^2^- to 10^3^- fold compared to CEF group. Until about 24 h later, the differences of the population in MAG+CEF group and CEF group reached 10^4^- to 10^6^- fold. Moreover, the bactericidal effect appeared to be dose-dependent, as demonstrated by the phenomenon that the group with equal level of cefquinome but with a high level of magnolol exhibited clearly stronger bactericidal activity than the low level of magnolol group, which indicated that magnolol may be a potential antibiotic activator. The above results suggest that magnolol exerts an effective and rapid bactericidal effect on MDR *E. coli*.

### 3.5. Drug-resistance curve analyses

**Figure 5:**
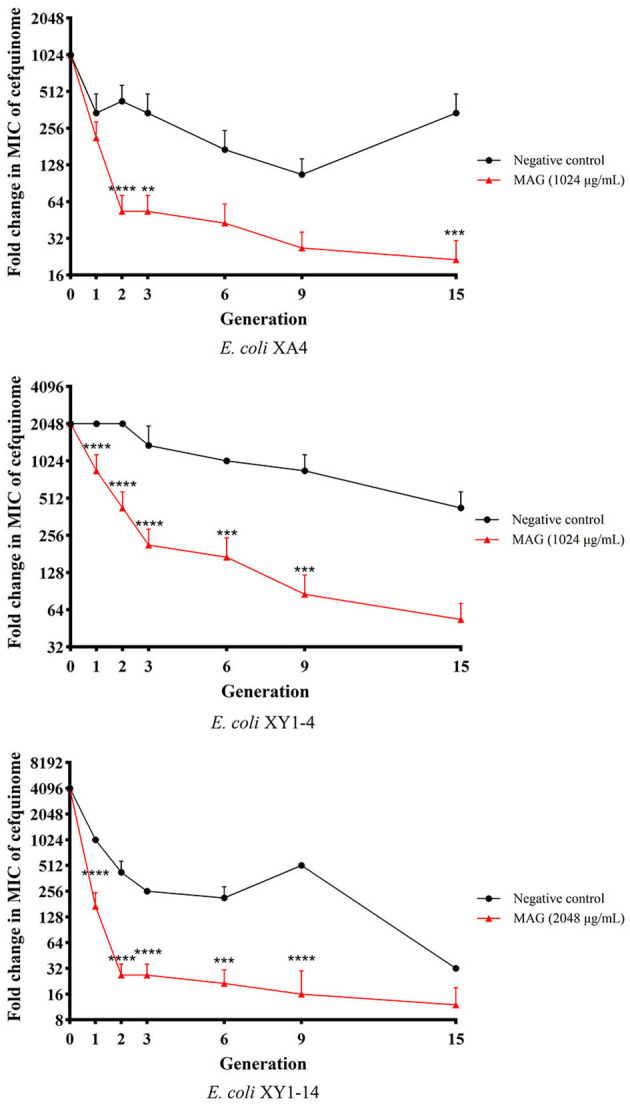
Effect of magnolol (MAG) on changing the sensitivity of MDR *E. coli* to cefquinome. All data are expressed as mean ± SD determined from three independent experiments performed in triplicate. ^ns^*P* > 0.05; **P < 0.01; ***P < 0.001; ****P < 0.0001.

Based on the results of MICs determined in generations 0, 1, 2, 3, 6, 9, and 15, drug-resistance curves were prepared to evaluate the changes in drug resistance of MDR *E. coli* treated with magnolol over the course of 15 generations. As shown in Figure 5, compared with the negative control group, the MICs of MDR *E. coli* strains in the magnolol group decreased more quickly in the first two generations (4- to 8- fold compared to the negative group). Starting with generation three, the MICs in the magnolol (MAG) group continued to decrease, and the changes in MICs were more stable than those of the negative control group, suggesting that magnolol is a strong and stable synergistic activator of cefquinome. After 15 generations, the MIC values in the magnolol group were 16 times lower than the negative control group in average. Regardless of the generation, except the generation 1 of the *E. coli* XA4, there were statistically significant differences between the negative control group and the MAG group, as the standard that: ^*^P < 0.05 (difference), ^**^P < 0.01 (significant difference), ^***^P < 0.001 (significant difference), ^****^P < 0.0001 (significant difference). These results collectively suggested that magnolol increases the sensitivity of MDR *E. coli* to cefquinome.

## 4. Discussion

The emergence of MDR *E. coli* has become a worldwide public health concern (28). Numerous surveillance studies of MDR bacteria in clinical and veterinary medicine have demonstrated that MDR *E. coli* is associated with an increased risk of transmission and poses a significant threat to the sale of food products and public health (29–31). To understand the current situation of MDR *E. coli*, we examined the presence of 22 drug-resistance genes in 101 *E. coli* strains isolated from eight cities in China, including Yulin, Yan'an, Shangluo, Xianyang, Xi'an, Hanzhong, Weinan, and Baoji.

The results of this study showed that the rates of MDR *E. coli* and the numbers and kinds of antibiotic-resistance genes carried by the isolates varied in the different cities. For example, the MDR rate in Weinan was 0.00%, but the rates in other cities were over 50%. Moreover, no strains carrying more than 10 antibiotic-resistance genes were found in Shangluo and Weinan. However, the rates in Yulin and Yan'an were over 20%. The question then arises: what could cause such a phenomenon? After communicating with local veterinarians, we hypothesized that the variations may be related to local and individual medication habits. For instance, veterinarians in Weinan preferred natural extracts to antibiotics when treating bacterial infections; thus, the rate of MDR *E. coli* was lowest in Weinan. This factor is related not only to the MDR *E. coli* rate but also the prevalence of antibiotic-resistance genes. Veterinarians in Xianyang and Xi'an used more aminoglycosides than β-lactams; therefore, the number of aminoglycoside-resistance genes carried by the isolates was greater than the number of β-lactam–resistance genes, and the same case can be found in the MIC results.

However, the prevalence of antibiotic resistance cannot be related to just local and individual medication habits. To examine the issue further, the results of this study were compared with those of previous studies in China. In the case of extended-spectrum β-lactamase (ESBL)-resistant *E. coli*, the rate of *bla*_*CTX*−*M*_in Yangzhou city was higher than the rates in the previous study (32). Furthermore, the differences were not only in rates but also gene types. In our study, the *bla*_*CTX*−*M*_ genes detected included *bla*_*CTX*−*M*−1_and *bla*_*CTX*−*M*−9_, but *bla*_*CTX*−*M*−14_, *bla*_*CTX*−*M*−15_, and *bla*_*CTX*−*M*−55_ were detected in Yangzhou city. Although *bla*_*CTX*−*M*−1_ and *bla*_*CTX*−*M*−15_, *bla*_*CTX*−*M*−9_, and *bla*_*CTX*−*M*−14_ belong to the same gene group, some differences still exists in the molecular structures, which led us to a second hypothesis, that the prevalence of antibiotic-resistance genes may be related to geographic factors that affect the molecular characteristics of the genes. This hypothesis can be verified in northeastern China (33).

Public policy is another factor that could affect the prevalence of antibiotic resistance. In 2015, the European Union published guidelines on the prudent use of antimicrobial veterinary medicines, the implementation of which led to lower rates of MDR strains in Europe (34–37) compared with China, where appeals to reduce and replace antibiotics with other agents in veterinary medicine did not occur until 2020. The degree of social and economic development can also affect the prevalence of antibiotic resistance. Compared with previous studies in Africa and west Asia (38–40), the rates of antibiotic-resistance genes in this study were lower, except for *bla*_*TEM*−1_. The impact of social and economic development can also be verified through comparisons with wealthier regions. The rates of ESBL-resistance genes in this study were slightly higher than the rates in United States (41, 42).

In summary, four factors can affect the prevalence of canine-origin antibiotic-resistant *E. coli*: 1. Local and individual medication habits; 2. Geographic factors that impact molecular characteristics of the genes; 3. Differences in public policies; and 4. The degree of social and economic development.

With the development of high-grade cephalosporins, resistance to these agents has also developed, which in turn has led to the emergence of ESBL-resistant *E. coli*. Resistance to cefquinome, the highest grade of cephalosporin used in veterinary medicine, has also emerged. Therefore, the identification of potent adjuvants to rescue cefquinome activity is of high priority. Currently, most studies examining the synergistic effects of combinations of natural compounds and antibiotics focus on inhibiting bacterial growth. A previous study found that resveratrol combined with colistin showed synergistic effects against *E. coli* (43). Another study found that the combination of salicylate and curcumin can inhibit colistin-resistant *E. coli* by inhibiting efflux pumps (44). Zhou et al. (45) identified pterostilbene as a potential MCR-1 inhibitor, which when combined with polymyxin B showed synergistic effects against *E. coli*. Studies of the efficacy of combinations of natural extracts and antibiotics against bacterial infections are not limited to *E. coli*. Cai et al. (46) reported that baicalin inhibits the *CTX-M-1* gene and that the combination of baicalin and cefotaxime shows synergistic effects against *Klebsiella pneumoniae*. Yi et al. (47) reported the synergistic antibacterial activity of tetrandrine combined with colistin against MCR-mediated colistin-resistant *Salmonella*.

Although magnolol has multiple biological activities, including the prevention and amelioration of diseases such as cancer (13), anti-depressant (14) and anti-diabetes (15) effects, and improvement of growth performance (16), its potential activity against bacterial diseases has not been fully explored. In this study, we unexpectedly found that magnolol exhibits potent potentiation (8- to 64-fold) of the effectiveness of cefquinome against resistant bacteria, even at high magnolol concentrations. To our knowledge, this study is the first to report the effect of the combination of magnolol and cefquinome in inhibiting cefquinome-resistant bacteria. Importantly, based on previous studies and the reported safety and toxicology of magnolol (19), we evaluated high levels of magnolol with cefquinome to determine if the bactericidal effect is related to magnolol dose. As indicated by time-kill curve analyses, the bactericidal effect was magnolol dose dependent. The above results clearly indicate that continuous combination therapy in relatively low doses (1/4 MIC to 1/2 MIC) is needed to effectively reduce resistance to cefquinome, suggesting that it is possible to reduce antibiotic resistance through continuous use of combinations of natural extracts and antibiotics. To predict the possibility of the use of magnolol in veterinary clinic against bacterial infection, several previous studies were referred. First of all, the blood concentration of magnolol can reach 0.74 μg/mL (ppm) in rats (19, 48) indicating that it's hard to achieve bactericidal concentrations in *vivo*. What's more, the literature and studies about the effects of magnolol against bacterial infection in *vivo* are very rare, including studies of effective physiological concentrations of magnolol and the mechanism of magnolol against bacterial infection *in vivo*. However, it's a common sense that the internal environment is complex and changeable. No matter whether cells or body is infected with germs, the system of innate immune response (TLR4/NF κB p65 pathway) will be activated promptly which will contribute to inflammation to remove the pathogens (49, 50). Therefore, the effects of inflammation and oxidative damage should be taken into account while evaluating the anti-bacterial effects of magnolol in *vivo*. As a result, relevant studies remain to be conducted.

As previous studies have indicated, antibacterial drug combinations can exhibit synergism in four ways: elimination of drug-resistance plasmids (51), inhibition of biofilm formation (52), inhibition of the activity of drug-resistance enzymes (45), and inhibition of drug efflux pumps (53). Based on previous research (18), we speculate that the mechanism of the synergy observed in this study is related to the inhibition of β-lactamases.

This study has some limitations. First, the sample size in the different cities was relatively small; thus, larger samples will be needed to verify the four above-mentioned hypotheses. Second, despite the observed synergism of the combination of magnolol and cefquinome, the mechanism of the synergy remains to be explored. Transcriptome sequencing has been used in drug-resistance reduction studies (27, 54) to identify changes in the expression of drug-resistance genes, data which can in turn be used in further studies to elucidate the effect of magnolol on the expression of drug-resistance genes. Furthermore, molecular docking and Western blotting have been used to evaluate the inhibitory effects of drugs on the activity and expression of drug-resistance enzymes (55, 56). These techniques can also be used to determine the inhibitory effect of magnolol on the activity and expression of ESBLs.

In conclusion, our data indicate that MDR *E. coli* strains are present in high rates in dogs kept as pets in China, thus raising public health concerns. Our study also demonstrated the synergistic effects of magnolol combined with cefquinome against MDR *E. coli*. However, the mechanism of this synergistic activity remains to be elucidated in our further studies. The discovery of magnolol as a novel cefquinome adjuvant highlights the enormous antibacterial potential of compounds extracted from plants.

## Data availability statement

The original contributions presented in the study are included in the article/Supplementary material, further inquiries can be directed to the corresponding authors.

## Ethics statement

The animal study was reviewed and approved by the Institutional Animal Care and Use Committee and Ethics Committee of Northwest A&F University (approval number: NWLA-2021-063). Written informed consent was obtained from the owners for the use of their animals in this study.

## Author contributions

Y-CT and Y-NZ conceived and designed the experiments, analyzed the data, and wrote the manuscript. Y-CT, Y-NZ, P-CL, Y-LC, D-ZD, YY, Q-YL, and Y-NG performed the experiments. S-ZQ interpreted the study results. W-RM and W-MZ revised the manuscript. All authors contributed to the manuscript and approved the submitted version.

## Supplementary material

Supplementary Figure 1 **(A)** Detection of drug-resistance genes carried by 101 *E. coli* stains identified. **(B)** MICs of 101 identified *E. coli* strains for 10 antibiotics.

Supplementary Table 1 Details and background of dogs from which samples were collected. ^*^S N., Sample Number; ^a^GR, Golden Retriever; ^b^CRD, Chinese Rural Dog; ^c^CS, Cocker Spaniel.

Supplementary Table 2 Sequences of *E. coli* drug resistance–related gene primers used in PCR assays.
