# Supplementary figures and images for: Detection of antibiotic-resistant canine origin Escherichia coli and the synergistic effect of magnolol in reducing the resistance of multidrug-resistant Escherichia coli
Source: Front Vet Sci. 2023 Mar 15;10:1104812. doi: 10.3389/fvets.2023.1104812 (PMC10057116; doi:10.3389/fvets.2023.1104812)

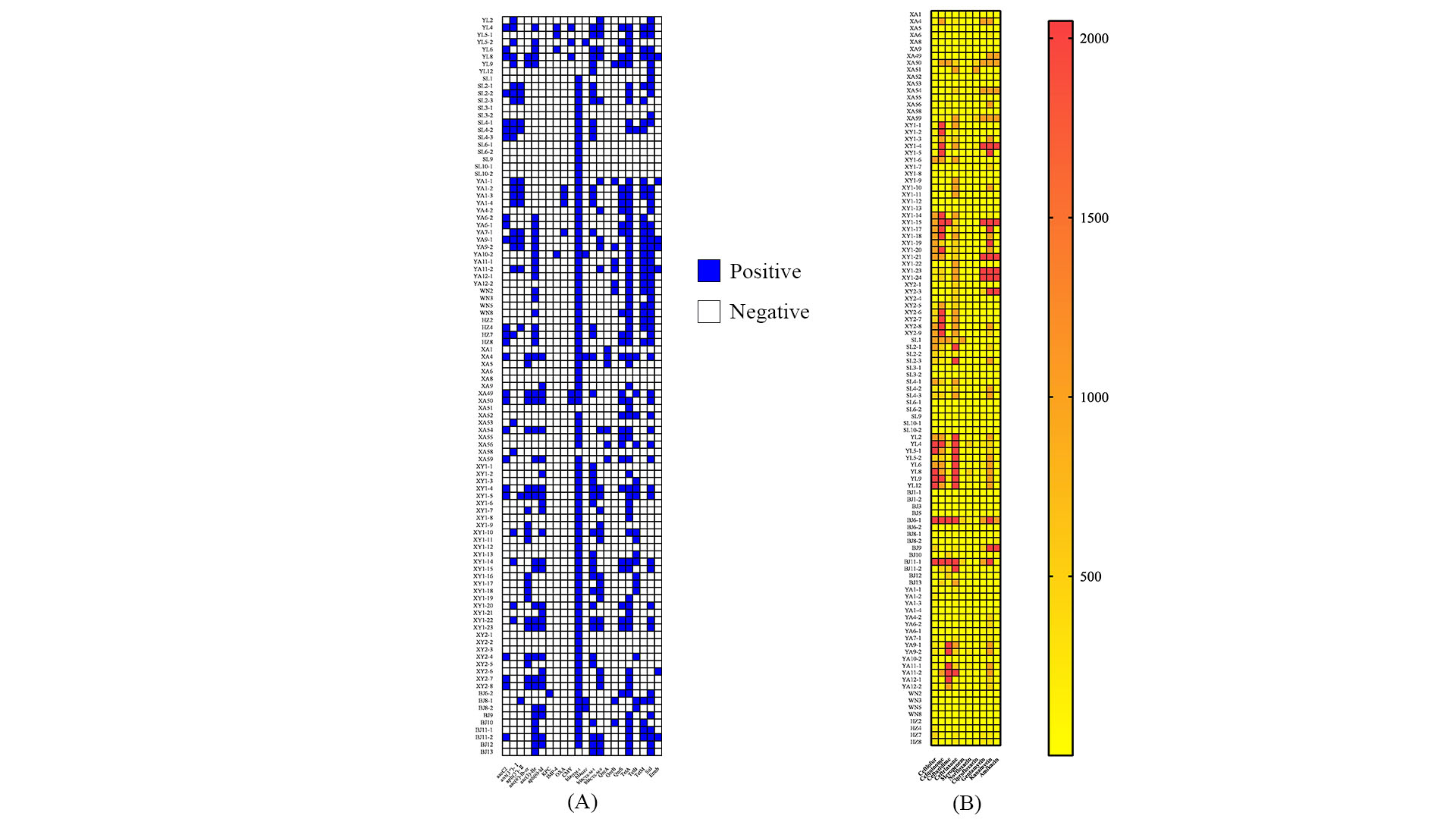

Supplement: Supplementary Figure 1 — (A) Detection of drug-resistance genes carried by 101 E. coli stains identified. (B) MICs of 101 identified E. coli strains for 10 antibiotics. [file Image_1.JPEG]
