# Supplementary material for: Detection of antibiotic-resistant canine origin Escherichia coli and the synergistic effect of magnolol in reducing the resistance of multidrug-resistant Escherichia coli
Source: Front Vet Sci. 2023 Mar 15;10:1104812. doi: 10.3389/fvets.2023.1104812 (PMC10057116; doi:10.3389/fvets.2023.1104812)
Supplement: Supplementary Table 1 — Details and background of dogs from which samples were collected. *S N., Sample Number; aGR, Golden Retriever; bCRD, Chinese Rural Dog; cCS, Cocker Spaniel. [file Table_1.DOCX]

**Table S1** Details and background of dogs from which samples were collected

| **S N.*** | **Breed** | **Age** | **S N.*** | **Breed** | **Age** | **S N.*** | **Breed** | **Age** | **S N.*** | **Breed** | **Age** | **S N.*** | **Breed** | **Age** | **S N.*** | **Breed** | **Age** | **S N.*** | **Breed** | **Age** |
| --- | --- | --- | --- | --- | --- | --- | --- | --- | --- | --- | --- | --- | --- | --- | --- | --- | --- | --- | --- | --- |
| YL1 | Husky | 2 | SL6-1 | Hiromi | 3 | YA12-1 | Husky | 4 | HZ9-2 | Hiromi | 2 | XY1-1 | CRD^b^ | 5 | XY1-26 | GR^a^ | 3 | BJ8-1 | CS^c^ | 3 |
| YL2 | Labrador | 3 | SL6-2 | Poodle | 5 | YA12-2 | Teddy | 4 | HZ10 | Samoye | 3 | XY1-2 | CRD^b^ | 2 | XY1-27 | CRD^b^ | 3 | BJ8-2 | Alaskan | 4 |
| YL3 | Labrador | 3 | SL7 | Samoye | 4 | YA13-1 | CRD^b^ | 5 | HZ11 | Husky | 3 | XY1-3 | Poodle | 3 | XY1-28 | CRD^b^ | 4 | BJ9 | GR^a^ | 5 |
| YL4 | Samoye | 4 | SL8 | Labrador | 3 | YA13-2 | CRD^b^ | 5 | XA1 | Poodle | 2 | XY1-4 | Husky | 2 | XY2-1 | GR^a^ | 2 | BJ10 | CRD^b^ | 4 |
| YL5-1 | GR^a^ | 2 | SL9 | Labrador | 5 | WN1 | CRD^b^ | 3 | XA2 | GR^a^ | 2 | XY1-5 | Husky | 4 | XY2-2 | Husky | 3 | BJ11-1 | Teddy | 6 |
| YL5-2 | Teddy | 5 | SL10-1 | GR^a^ | 6 | WN2 | GR^a^ | 4 | XA3 | Corgi | 4 | XY1-6 | Labrador | 5 | XY2-3 | Alaskan | 6 | BJ11-2 | GR^a^ | 5 |
| YL6 | Teddy | 6 | SL10-2 | Poodle | 5 | WN3 | Shiba Inu | 5 | XA4 | CRD^b^ | 4 | XY1-7 | Corgi | 2 | XY2-4 | Teddy | 5 | BJ12 | Husky | 5 |
| YL7 | Du Bin | 3 | SL11-1 | Corgi | 3 | WN4 | Poodle | 6 | XA5 | GR^a^ | 3 | XY1-8 | Alaskan | 5 | XY2-5 | Labrador | 3 | BJ13 | Hiromi | 3 |
| YL8 | CRD^b^ | 3 | SL11-2 | Poodle | 2 | WN5 | Labrador | 2 | XA6 | CRD^b^ | 2 | XY1-9 | Corgi | 2 | XY2-6 | Husky | 2 |  |  |  |
| YL9 | CRD^b^ | 4 | YA1-1 | CRD^b^ | 2 | WN6 | CS^c^ | 2 | XA7 | GR^a^ | 4 | XY1-10 | CRD^b^ | 3 | XY2-7 | Samoye | 4 |  |  |  |
| YL10 | CRD^b^ | 2 | YA1-2 | Samoye | 3 | WN7 | GR^a^ | 3 | XA8 | CRD^b^ | 3 | XY1-11 | CRD^b^ | 6 | XY2-8 | Corgi | 5 |  |  |  |
| YL11 | Shiba Inu | 3 | YA1-3 | CRD^b^ | 6 | WN8 | Poodle | 3 | XA9 | Teddy | 4 | XY1-12 | GR^a^ | 3 | XY2-9 | Corgi | 3 |  |  |  |
| YL12 | Shiba Inu | 4 | YA1-4 | GR^a^ | 5 | WN9 | CRD^b^ | 4 | XA49 | Teddy | 4 | XY1-13 | Hiromi | 5 | XY2-10 | CRDb | 2 |  |  |  |
| YL13 | Poodle | 2 | YA2 | Corgi | **3** | WN10 | CRD^b^ | 4 | XA50 | Shiba Inu | 6 | XY1-14 | GR^a^ | 5 | XY2-11 | GR^a^ | 6 |  |  |  |
| YL14 | GR^a^ | 5 | YA3 | Corgi | **3** | WN11 | Husky | 3 | XA51 | Hiromi | 3 | XY1-15 | Labrador | 5 | XY2-12 | Hiromi | 3 |  |  |  |
| SL1 | CRD^b^ | 3 | YA4-1 | Labrador | 3 | WN12 | Hiromi | 2 | XA52 | Labrador | 4 | XY1-16 | Hiromi | 4 | XY2-13 | Labrador | 4 |  |  |  |
| SL2-1 | CRD^b^ | 3 | YA4-2 | Du Bin | 5 | HZ1 | Samoye | 2 | XA53 | Husky | 5 | XY1-17 | Samoyer | 4 | XY2-14 | Shiba Inu | 5 |  |  |  |
| SL2-2 | CRD^b^ | 4 | YA5 | Corgi | 6 | HZ2 | Husky | 3 | XA54 | Labrador | 6 | XY1-18 | Poodle | 6 | BJ1 | Labrador | 3 |  |  |  |
| SL2-3 | Corgi | 5 | YA6-1 | Samoye | 4 | HZ3 | Alaskan | 5 | XA55 | CS^c^ | 3 | XY1-19 | CRD^b^ | 3 | BJ2 | Hiromi | 3 |  |  |  |
| SL3-1 | Husky | 6 | YA6-2 | GR^a^ | 4 | HZ4 | Labrador | 2 | XA56 | CRD^b^ | 2 | XY1-20 | Teddy | 2 | BJ3 | Labrador | 6 |  |  |  |
| SL3-2 | Alaskan | 6 | YA7 | GR^a^ | 3 | HZ5 | Labrador | 3 | XA57 | CRD^b^ | 3 | XY1-21 | CRD^b^ | 3 | BJ4-1 | CRD^b^ | 3 |  |  |  |
| SL4-1 | Husky | 3 | YA8 | CRD^b^ | 3 | HZ6 | CRD^b^ | 4 | XA58 | Du Bin | 3 | XY1-22 | CRD^b^ | 3 | BJ4-2 | CRD^b^ | 2 |  |  |  |
| SL4-2 | CRD^b^ | 2 | YA9-1 | CRD^b^ | 4 | HZ7 | CRD^b^ | 5 | XA59 | Teddy | 3 | XY1-23 | CRD^b^ | 3 | BJ5 | Corgi | 5 |  |  |  |
| SL4-3 | CRD^b^ | 2 | YA9-2 | CRD^b^ | 2 | HZ8 | CRD^b^ | 5 | XA51 | Hiromi | 3 | XY1-24 | Corgi | 5 | BJ6-1 | Husky | 3 |  |  |  |
| SL5 | Teddy | 3 | YA10-1 | CRD^b^ | 5 | HZ9-1 | Labrador | 3 | XA52 | Labrador | 4 | XY1-25 | CRD^b^ | 6 | BJ6-2 | Shiba Inu | 2 |  | | |

*** S N.: Sample Number**

**^a^ GR: Golden Retriever**

**^b^ CRD: Chinese Rural Dog**

**^c^ CS: Cocker Spaniel**
