# Supplementary material for: Detection of antibiotic-resistant canine origin Escherichia coli and the synergistic effect of magnolol in reducing the resistance of multidrug-resistant Escherichia coli
Source: Front Vet Sci. 2023 Mar 15;10:1104812. doi: 10.3389/fvets.2023.1104812 (PMC10057116; doi:10.3389/fvets.2023.1104812)
Supplement: Supplementary Table 2 — Sequences of E. coli drug resistance–related gene primers used in PCR assays. [file Table_2.DOCX]

**Table S2** Sequences of *E. coli* drug resistance–related gene primers used in PCR assays

| Gene | Sequence of primer (5’~3’) | Size of product/bp | Tm/°C |
| --- | --- | --- | --- |
| *aacC2* | F:ATGCATACGCGGAAGGCAAT | 861 | 57.4 |
|  | R:CTAACCTGAAGGCTCGCAAG |  |  |
| *ant(3’)-Ⅰ* | F:TAAACATCATGAGGGAAGCG | 803 | 53.4 |
|  | R:ACATTATTTGCCGACTACCT |  |  |
| *aph(3’)-Ⅱ* | F:TCTGAAACATGGCAAAGGTAG | 582 | 53.7 |
|  | R:AGCCGTTTCTGTAATGAAGGA |  |  |
| *aac(6’)-Ⅰb-cr* | F:AATCGATCTCATATCGTCGAGTGG | 214 | 57.9 |
|  | R:GTACTCCTGGATCGGTTTCTTCTT |  |  |
| *aac(3’)-Ⅱe* | F:GTTCGGCCTGCTGAATCAATTT | 304 | 57.7 |
|  | R:GACTTCACCGTTTCTTCCAAGC |  |  |
| *bla_KPC_* | F:TCCCACTGTGCAGCTCATTC | 293 | 60.3 |
|  | R:GTCCAGACGGAACGTGGTAT |  |  |
| *bla_IMP-4_* | F:CAATCCATCCCCACGTATGC | 346 | 58.7 |
|  | R:TCAAGAGTGATGCGTCTCCA |  |  |
| *bla_OXA_* | F:AATTGGCGAGGCACGTATGA | 284 | 60.1 |
|  | R:CGACCCACCAGCCAATCTTA |  |  |
| *bla_CMY_* | F:GGAATGAGTTACGAAGAGGCAATG | 315 | 59.7 |
|  | R:CCAGCCTAATCCCTGGTACATATC |  |  |
| *bla_TEM-1_* | F: TTCGTGTCGCCCTTATTC | 820 | 57.9 |
|  | R: CCTATCTCAGCGATCTGTCTA |  |  |
| *bla_SHV_* | F:GCCATTACCATGAGCGATAACAG | 423 | 59.6 |
|  | R:GGTATCCCGCAGATAAATCACCAC |  |  |
| *bla_CTX-M-1_* | F:GGTGACATGGATGAAAGGCAATAC | 175 | 57.9 |
|  | R:CTGGGTGAAGTAAGTGACCAGAAT |  |  |
| *bla_CTX-M-9_* | F:CTCATCGATACCGCAGATAATACG | 652 | 57.9 |
|  | R:GCTGGGTAAAATAGGTCACCAGAA |  |  |
| *Qnra* | F:ATCTCAAGGGCGCCAACTTT | 327 | 60.2 |
|  | R:AGGCACAGATCTTGACTCCTT |  |  |
| *Qnrb* | F:AGTCGTGCGATGCTGAAAGA | 333 | 60.0 |
|  | R:TCGCCAGTCGAAAGTCGAAA |  |  |
| *Qnrs* | F:GTCGCAGATCTTCGTGATGC | 286 | 59.4 |
|  | R:CTTTCAGTGATGCACCCGCT |  |  |

| *TetA* | F:CAGCCTTGAACGGCCTCAAT | 290 | 60.9 |
| --- | --- | --- | --- |
|  | R:CTGGGCGAGTGAATGCAGAA |  |  |
| *TetB* | F:GCAACGGTGTGGGTGCTATT | 257 | 61.2 |
|  | R:ATCCCACCACCAGCCAATAA |  |  |
| *TetM* | F:AACGACTGTTGAACCGAGCA | 416 | 60.1 |
|  | R:CGCAACCATAGCGTATCCCT |  |  |
| *Ermb* | F:AAAGCCATGCGTCTGACATCT | 204 | 60.3 |
|  | R:CTGGAACATCTGTGGTATGGC |  |  |
| 16s rDNA | F:AGAGTTTGATCCTGGCTCAG | 306 | 55.0 |
|  | R: CTTGTGCGGGCCCCCGTCAATTC |  |  |
